# Extending Asia Pacific bioinformatics into new realms in the "-omics" era

**DOI:** 10.1186/1471-2164-10-S3-S1

**Published:** 2009-12-03

**Authors:** Shoba Ranganathan, Frank Eisenhaber, Joo Chuan Tong, Tin Wee Tan

**Affiliations:** 1 Department of Chemistry and Biomolecular Sciences and ARC Centre of Excellence in Bioinformatics, Macquarie University, Sydney NSW 2109, Australia; 2 Department of Biochemistry, Yong Loo Lin School of Medicine, National University of Singapore, 8 Medical Drive, Singapore 117597; 3 Bioinformatics Institute, 30 Biopolis Street; #07-01 Matrix, Singapore; 4 Institute for Infocomm Research, 1 Fusionopolis Way, #21-01 Connexis, Singapore

## Abstract

The 2009 annual conference of the Asia Pacific Bioinformatics Network (APBioNet), Asia's oldest bioinformatics organisation dating back to 1998, was organized as the 8^th ^International Conference on Bioinformatics (InCoB), Sept. 7-11, 2009 at Biopolis, Singapore. Besides bringing together scientists from the field of bioinformatics in this region, InCoB has actively engaged clinicians and researchers from the area of systems biology, to facilitate greater synergy between these two groups. InCoB2009 followed on from a series of successful annual events in Bangkok (Thailand), Penang (Malaysia), Auckland (New Zealand), Busan (South Korea), New Delhi (India), Hong Kong and Taipei (Taiwan), with InCoB2010 scheduled to be held in Tokyo, Japan, Sept. 26-28, 2010. The Workshop on Education in Bioinformatics and Computational Biology (WEBCB) and symposia on Clinical Bioinformatics (CBAS), the Singapore Symposium on Computational Biology (SYMBIO) and training tutorials were scheduled prior to the scientific meeting, and provided ample opportunity for in-depth learning and special interest meetings for educators, clinicians and students. We provide a brief overview of the peer-reviewed bioinformatics manuscripts accepted for publication in this supplement, grouped into thematic areas. In order to facilitate scientific reproducibility and accountability, we have, for the first time, introduced minimum information criteria for our pubilcations, including compliance to a Minimum Information about a Bioinformatics Investigation (MIABi).  As the regional research expertise in bioinformatics matures, we have delineated a minimum set of bioinformatics skills required for addressing the computational challenges of the "-omics" era.

## Overview

The Asia-Pacific Bioinformatics Network (APBioNet, [1]) is the oldest regional bioinformatics society, established in 1998, to gather scientists from diverse disciplines to work together to advance the frontiers of bioinformatics. The first three annual meetings were held at the Pacific Symposium of Biocomputing (1999-2001), in Hawaii, to nucleate a bioinformatics core group in the region. With the core group reaching critical mass, the APBioNet executive committee members assisted in organizing InCoB2002 (the International Conference on Bioinformatics, 2002) in Bangkok, Thailand and adopted this meeting as their annual conference. Following this successful event, InCoB meetings have been held in Penang, Malaysia (2003); Auckland, New Zealand (2004); Busan, South Korea (2005), New Delhi, India (2006); Hong Kong/Hanoi (by videocasting) (2007) and Taipei, Taiwan (2008, [2]).

In order to sustain the growth of bioinformatics in the region, a special interest group meeting of bioinformatics educators, the Workshop on Education in Bioinformatics and Computational Biology (WEBCB) was organized at InCoB2008, attended also by life scientists participating in the symposium of the Federation of Asian and Oceanician Biochemists and Molecular Biologists (FAOBMB) at the same location. WEBCB follows on the Workshops on Education in Bioinformatics (WEB, initiated by SR) [3], to incorporate bioinformatics into mainstream life science research and its second meeting was held at InCoB2009, along with tutorials in traditional and emerging topics as part of APBioNet's training mandate.

The first ever Clinical Bioinformatics Symposium (CBAS) was held prior to the InCoB2009 scientific conference, while the Singapore chapter of the Regional Student Group of the International Society for Computational Biology organized the Singapore Symposium on Computational Biology (SYMBIO) to provide students and young investigators an opportunity to present their ongoing research work.

In order to provide opportunities for international peer-reviewed high-impact factor journal publications, APBioNet embarked on further raising the standards for the region by publishing a dedicated *BMC Bioinformatics* supplement, since 2006 [2,4,5]. In 2009, the manuscripts from APBioNet members have diversified and increased in quality and sophistication with computational biology articles addressing analysis pertaining to "-omics" data published in this supplement and a *BMC Bioinformatics* supplement [6], focussing on protein sequence analysis, genetic and population analysis, structural bioinformatics, text mining and ontology, chemoinformatics and biodiversity informatics as well as a case study on the impact of e-learning tools in bioinformatics education.

## Reviewing standards

Papers submitted to these proceedings were peer-reviewed by at least three reviewers, from the APBioNet/InCoB program committee and invited external experts as required (listed in Additional File 1). InCoB2009 also provided multi-track submissions, with the inclusion of research highlights from recent publications and for showcasing technology developments along with posters. With tutorials, a specialist workshop on bioinformatics education and two special interest research symposia, InCoB2009 provided a comprehensive five-day international bioinformatics meeting in the Asia Pacific.

The editors carefully screened the 90 full paper submissions and relegated two of these to the poster session, in order to select only the best papers from more than a dozen Asia Pacific countries, as well as UK and USA. From the 88 full paper submissions reviewed, 49 were short listed for oral presentation. This supplement features 34 papers addressing "-omics" data analysis, while 10 papers were accepted for publication in the BMC Bioinformatics supplement [6], reflecting an overall acceptance rate of 50%, with five more appearing in the online journal, *Bioinformation *[7]. Extensive collaboration in bioinformatics research in the region is evident from co-authorship of papers involving Australia, China, Hong Kong, India, Japan, Korea, Singapore, Taiwan, Thailand, UK and USA. A brief review of the different themes is provided below.

## Next-generation sequence analysis

With the advent of next-generation sequencing technologies, nucleotide sequencing has become inexpensive, albeit resulting in short sequence reads, requiring new and efficient bioinformatics tools. Zhao et al. [8] present an alignment tool, BOAT, capable of mapping large volumes of short reads to reference sequences with better sensitivity and lower memory requirement than other currently existing algorithms. Chung and Park [9] propose an empirical method for choosing efficient discriminative seeds for oligonucleotide design, while Piriyapongsa et al. [10] have developed a new integrated primer design tool. Venkatachalam et al. [11] have predicted the occurrence of peroxisome proliferator response elements, which are promising targets for cancer treatment, followed by *in vitro *experimental validation. In order to accurately predict the caspase degradome on a systems-wide basis, Wee et al. [12] have developed a multifactor model incorporating cleavage site prediction and structural factors.

## Genome analysis

To track genome-wide transposon-based insertional mutagenesis experiments, Yang et al. [13] have developed MP-PBmice, a web-based application for large-scale insertional mutation mapping onto the mouse genome, while Lim et al. [14] present BioBarcode, a database resource for Asian biodiversity resources. Jenjaroenpun and Kuznetsov [15] tackle the problem of identifying triplex DNA forming region, towards discovering biologically meaningful genome modules and to optimize experimental design of anti-gene treatments, while Saeed and Halgamuge [16] propose a one-dimensional signature derived from oligonucleotide frequency, for efficient grouping or "binning" of metagenome fragments, crucial to the success of microbial genome consortia. Chacko and Ranganathan [17] present the genome-wide analysis of alternative splicing in the bovine genome, with implications for using the cow as a model for specific human diseases.

## Genetic and population analysis

For genotype-phenotype correlations, understanding mitochondrial variations of each individual, haplogroup or geographical location is of primary importance. MitoVariome (Lee at al. [18]) provides a human mitochondrial variation resource for researchers in this area. To identify splice variants and SNPs in short sequence reads from next-generation sequencing approaches, Bao et al. [19] have developed a software tool, MapNext.

## Transcriptome analysis

DNA microarrays have led to a multitude of gene expression analysis studies. Nguyen and Lió [20] have developed a new metric, BayesGen, to measure the similarity between gene expression profiles, for constructing genome-wide co-expression networks, and for clustering cancer human tissues into subtypes. Kuem et al. [21] present a pathway-based gene expression similarity measure, which outperforms other commonly used similarity measures, while Ho et al. [22] have used quantile regression models to characterize gene-expression patterns underpinning the molecular regulatory mechanisms leading to mammalian ageing.

## Interactome analysis

Interaction data from biology present enormous challenges to bioinformatics in the "-omics" era and contribute to our understanding the functions of biological molecules and systems. Kadupitige et al. [23] present a tool for exploratory analysis of gene interaction networks, while Park et al. [24] have developed a prototype database and server for analyzing protein interaction networks. Maulik et al. [25] have applied protein interaction data to understand plant defense mechanisms, while Reja et al. [26] have developed MitoInteractome, a mitochondrial protein interaction resource for characterizing the ageing process.

## Structural genomics

Towards understanding biological function, large-scale biological structure determination projects are currently underway, leading to structure analysis into protein domains by Yoo et al. [27]. Where experimental structures are not available, structure prediction is still an important area of research, with Liu et al. [28] presenting a new sequence-based hybrid predictor to identify conformationally ambivalent regions in proteins. Huang et al. [29] have used sequence and structural information to predict DNA-binding residues in transcription factors, while Jongkon et al. [30] have predicted the binding preference of various strains of avian influenza A to cognate human receptors. Prasad et al. [31] have employed RNA secondary structure and sequence motifs for the phylogenetic analysis of flukes.

## Networks, pathways and systems biology

With the growing interest in systems biology, six studies in computations approaches to networks, pathways and systems were presented at InCoB2009. Kim and Gelenbe [32] propose the G-network for the steady-state behaviour of gene regulatory networks for identifying disease-causing genes while Le et al. [33] have applied rule induction learning to characterise nucleosome dynamics from genomic and epigenetic data. Chaturvedi and Rajapakse [34] have developed a skip-chain model to study time-delayed regulation in gene regulatory networks. Rhee et al. [35] identifed cell cycle-related regulatory motifs using a kernel canonical correlation analysis. Mapping virus-host protein interactions to host signalling pathways has been carried out [36] to characterize alternative pathways that can be targeted by drugs, while Min and Hong [37] has analysed how rate-limiting enzymes influence metabolic flux.

## Disease informatics

The human genome was primarily sequenced to understand the genetic basis of diseases. In this area, Yang et al. [38] have compiled a database of Parkinson's Disease-related genes and genetic variation. Khan and Ranganathan [39] have used a multi-species comparative structural approach of inherited mutations to correlate genotype to phenotype in an inherited disease.

## Clinical informatics

Ultimately, bioinformatics will have to process clinical data to address the quest for personalized medicine. As medical and biological data are commonly presented usually as small datasets without balanced class distribution, Yang et al. [40] have developed a particle swarm approach to address this imbalance for the discovery of new pathological conditions and disease subtypes. Jho et al. [41] have developed a data deposition system integrated with automated bioinformatics tools for mutation detection and analysis starting from raw patient data.

## Conclusion

Judging from the breadth and depth of topics covered in this issue, Asia Pacific bioinformatics research has not only maintained its level of research achievement, but extended computational approaches to systems biology and medical and clinical informatics. Computational biology is now acknowledged to be a core research discipline in our educational and research institutions. We also note that several papers bearing graduate students as first authors, testifying to the quality of research training imparted. In order to effectively train new entrants to undertake bioinformatics research in the "-omics" era, WEBCB has, over the past two years, run discussion fora to define the key elements constituting a minimum set of bioinformatics skills. Based on the outcome of these discussions, Tan et al. [42] present the minimum skill set in bioinformatics and computational biology that an ideal graduating student in life sciences would possess.

Furthermore, computational biology is now considered an essential area of research for supporting to "-omics" and health research, as reported by scientists from Malaysia [43] and Singapore [44] and elsewhere.  However, the situation is plagued by author ambiguity especially for Asian names, broken links for web tools, disappearing databases and inadequate disclosure, not enough for reproducibility.

New initiatives from the Asia-Pacific Bioinformatics network include compliance standards such as Minimum Information about a Bioinformatics investigation (MIABi), currently under development.  This MIABi compliance will require, firstly, for authors to be issued with unique author identifiers (e.g. see prototype at http://aid.apbionet.org/) for identity disambiguation and accountability purposes.  Authors with multiple identifiers issued by various publishers (e.g. Schopus author ID, researcherID) can now be resolved to a unique individual through indentifiers cross-referencing.

Secondly, it will eventually require deposition of scientific datasets through a central portal (e.g. http://docid.apbionet.org/) for persistence, provenanc, accessibility and reproducibility.  All databses, datasets and codes cited in papers published through our processes may be mandated to be archived in this way, supported by distributed repository nodes, such as that of the Asian Bioinformation Centers [45] initiative.  Moreover, a database on a pre-configured operating system (OS) such as BioSlax (http://www.bioslax.com) can also be archived as an image, stored at such repositories.  Even though the original database server may no longer be available, the database-OS image can be dynamically re-instantiated on demand via a cloud computing virtualized platform.

## Competing interests

The authors declare that they have no competing interests.

## Supplementary Material

Additional file 1 APBioNet InCoB2009 Program Committee members and reviewers. Click here for file
