# Supplementary material for: Extending Asia Pacific bioinformatics into new realms in the "-omics" era
Source: BMC Genomics. 2009 Dec 3;10(Suppl 3):S1. doi: 10.1186/1471-2164-10-S3-S1 (PMC2788361; doi:10.1186/1471-2164-10-S3-S1)
Supplement: Additional file 1 — APBioNet InCoB2009 Program Committee members and reviewers. [file 1471-2164-10-S3-S1-S1.pdf]

Extending Asia Pacific bioinformatics into new realms in the "-omics" era

Shoba Ranganathan, Frank Eisenhaber, Joo Chuan Tong and Tin Wee Tan

Additional File 1: APBioNet InCoB2009 Program committee members and invited expert reviewers

1. Shandar **AHMAD**, National Institute of Biomedical Innovation, Japan
2. Jonathan **ARTHUR**, University of Sydney, Australia
3. Vladimir **BAJIC**, King Abdullah University of Science and Technology, Saudi Arabia
4. Christopher **BAKER**, University of New Brunswick, Canada
5. Alex **BATEMAN**, The Wellcome Trust Sanger Institute, UK
6. Jong **BHAK**, Korean Bio Information Center (KOBIC), Korea
7. Michael **BODEN**, University of Queensland, Australia
8. Vladimir **BRUSIC,** Dana Faber Cancer Institute, USA
9. Yiyu **CAI**, Nanyang Technological University, Singapore
10. Zhiwei **CAO**, Shanghai Center for Bioinformation Technology, China
11. Elsa **CHACKO**, Macquarie University
12. Michael **CHARLESTON**, University of Sydney, Australia
13. Vijayalakshmi **CHELLIAH**, European Bioinformatics Institute, UK
14. Xin **CHEN**, Zhejiang University, China
15. Khar Heng **CHOO**, Institute for Infocomm Research, Singapore
16. Adrian **COOTES**, Macquarie University, Australia
17. Andew **DALBY**, University of Oxford, UK
18. Gloria **DESPACIO-REYES**, The Wellcome Trust Sanger Institute, UK
19. Jitendra **GAIKWAD**, Macquarie University, Australia
20. Ge **GAO**, Peking University, China
21. Christopher **HOGUE**, National University of Singapore, Singapore
22. Wen-Lian **HSU**, Academia Sinica, Taiwan
23. Zeti **HUSSEIN**, Universiti Kebangsaan Malaysia, Malaysia
24. Ming-Jing **HWANG**, Academia Sinica, Taiwan
25. Mohammad **ISLAM**, Macquarie University, Australia
26. Pandjassarame **KANGUEANE**, AIMST University, Malaysia
27. R. Krishna Murthy **KARUTURI**, Genome Institute of Singapore, Singapore
28. Asif **KHAN**, National University of Singapore, Singapore
29. Javed **KHAN**, Macquarie University, Australia
30. Varun **KHANNA**, Macquarie University, Australia
31. Judith **KLEIN-SEETHARAMAN**, University of Pittsburgh School of Medicine, USA
32. Gaurav **KUMAR**, Macquarie University, Australia
33. Igor **KUZNETSOV**, University of Albany, USA
34. Vladimir **KUZNETSOV,** Bioinformatics Institute, Singapore
35. Chee Keong **KWOH**, Nanyang Technological University, Singapore
36. Michael A. **LANGSTON**, University of Tennessee, USA
37. Bernett **LEE**, Eli Lilly, Singapore
38. Jinyan **LI**, Nanyang Technological University, Singapore
39. Vladimir **LIKIC**, University of Melbourne, Australia
40. Jingchu **LUO**, Peking University, China

41. Geoff **MCLACHLAN**, University of Queensland, Australia
42. Ranjeeta **MENON**, Macquarie University, Australia
43. Olivo **MIOTTO**, University of Oxford, UK
44. Satoru **MIYANO**, University of Tokyo, Japan
45. Shivashankar **NAGARAj**, CSIRO, Australia
46. H.A. **NAGARAJARAM**, Center for DNA Fingerprinting and Diagnostics (CDFD), India
47. Kenta **NAKAI**, University of Tokyo, Japan
48. Haruki **NAKAMURA**, Osaka University, Japan
49. Laszlo **ORBAN**, Temasek Life Sciences Laboratory, Singapore
50. Mohd Shahir Shamsir **OMAR**, University Teknologi Malaysia, Malaysia
51. Sujeevan **RATNASINGHAM**, University of Guelph, Canada
52. Toni **REVERTER**, CSIRO, Australia
53. Meena **SAKHARKAR**, Nanyang Technological University, Singapore
54. Bertil **SCHMIDT**, Nanyang Technology University, Singapore
55. Christian **SCHÖNBACH**, Kyushu Institute of Technology, Singapore
56. Amandeep **SIDHU**, Curtin University of Technology, Australia
57. D. **SUNDAR**, Indian Institute of Technology Delhi, India
58. R. **SOWDHAMINI**, National Centre for Biological Sciences, India
59. Subramanian **SUBBIAH**, Stanford University, USA
60. Prashanth **SURAVAJHALA**, Centre for Development of Advanced Computing, India
61. Antonius **SUWANTO**, Atma Jaya University, Indonesia
62. Daniel **SZE**, Hong Kong Polytechnic University, Hong Kong
63. Martti **TAMMI,** National University of Singapore, Singapore
64. Vivek **TANAVDE,** Bioinformatics Institute, Singapore
65. Rohan **TEASDALE**, University of Queensland, Australia
66. Joo Chuan **TONG**, Institute for Infocomm Research, Singapore
67. Sissades **TONGSIMA**, National Center for Genetic Engineering and Biotechnology, Thailand
68. Pascal **VALLOTTON**, CSIRO, Australia
69. Chandra **VERMA**, Bioinformatics Institute, Singapore
70. Lawrence **WEE**, National University of Singapore, Singapore
71. Kai **XU**, CSIRO, Australia
72. Hannah Hong **XUE**, Hong Kong University of Science and Technology, Hong Kong
73. Henry **YANG**, Singapore Immunology Network, Singapore
74. Ueng-Cheng **YANG**, National Yang-Ming University, Taiwan
75. Guang Lan **ZHANG**, Dana Farber Cancer Institute, USA
76. Louxin **ZHANG**, National University of Singapore, Singapore
77. Albert **ZOMAYA**, University of Sydney, Australia
